# Users' guide to the orthopaedic literature: What is a cost-effectiveness analysis?

**DOI:** 10.4103/0019-5413.40247

**Published:** 2008

**Authors:** Stephanie Tanner, Sheila Sprague, Kyle Jeray

**Affiliations:** Department of Orthopaedic Surgery, Greenville Hospital System, Greenville, South Carolina, USA; 1 Department of Clinical Epidemiology and Biostatistics, McMaster University, Hamilton, Ontario, Canada

**Keywords:** Cost-effectiveness, critical appraisal, evidence-based medicine, hierarchy of evidence

## Abstract

As the cost of healthcare continue to rise, orthopaedic surgeons are being pressured to practice cost-effective healthcare. Consequently, economic evaluation of treatment options are being reported more commonly in medical and surgical literature. As new orthopaedic procedures and treatments may improve patient outcome and function over traditional treatment options, the effect of the potentially higher costs of new treatments should be formally evaluated. Unfortunately, the resources available for healthcare spending are typically limited. Therefore, cost-effectiveness analyses have become an important and useful tool in informing which procedure or treatment to implement into practice.

Cost-effectiveness analysis is a type of economic analysis that compares both the clinical outcomes and the costs of new treatment options to current treatment options or standards of care. For a clinician to be able to apply the results of a cost-effectiveness analysis to their practice, they must be able to critically review the available literature. Conducting an economic analysis is a challenging process, which has resulted in a number of published economic analyses that are of lower quality and may be fraught with bias. It is important that the reader of an economic analysis or cost-effectiveness analysis have the skills required to properly evaluate and critically appraise the methodology used before applying the recommendations to their practice.

Using the principles of evidence-based medicine and the questions outlined in the Journal of the American Medical Association's Users' Guide to the Medical Literature, this article attempts to illustrate how to critically appraise a cost-effectiveness analysis in the orthopaedic surgery literature.

## INTRODUCTION

In a budget constrained healthcare systems, economic evaluations and specifically cost-effectiveness analyses are becoming more commonly reported in the orthopaedic literature.^1^ Many newer orthopaedic procedures and treatments promise improved patient outcomes and function over alternative treatment options, but at a significantly higher cost to the payer. There is currently a progressive limitation in the available resources to devote to healthcare in all countries.^2^ This problem will grow in the immediate future as healthcare needs are increasing more rapidly than the money that countries can spend in this area.^2^ For this reason, there is a growing demand by healthcare decision makers to have data on the efficacy and costs of new treatments.^2^

An economic analysis is a set of formal, quantitative methods used to compare alternative treatment strategies with respect to their resource use and their expected outcomes.^3^ A full economic analysis must consider both the costs and the outcomes or consequences of the alternative treatment options. The four types of economic analysis commonly reported in the medical and surgical literature are cost-minimization analysis, cost-effectiveness analysis, cost-utility analysis and cost-benefit analysis.^3^ This article will focus on the cost-effectiveness analysis and cost-utility analysis, which is a subset of cost-effectiveness analysis.

In a cost-effectiveness analysis, the consequences or health outcomes are expressed in natural units such as cost per unit of effect or in terms of the effect per unit of costs.^3^ For example, the units may be cost per life saved, cost per limb salvaged or costs per case successfully treated.^1^ Cost-effectiveness should be expressed as an incremental cost-effectiveness ratio (ICER), which is the ratio of change in costs to the change in effects.^3^ The numerator of the ICER is the marginal difference of the mean cost of each treatment option and the denominator is the marginal mean difference of the effectiveness of each treatment option.^3^

Cost-utility analysis is a type of cost-effectiveness analysis that presents the outcomes in terms of life-years adjusted by peoples' preferences. Typically, one considers the incremental cost per incremental gain in quality adjusted life-years (QALYs) which is calculated by adjusting the length of time affected by the health outcome by the utility value assigned to the resultant health status. Utility is usually expressed as a decimal from zero to one, with zero representing death and one representing perfect health.^3^ Utilities or preferences are global health related quality of life (HRQL) measures.^3^ There are several different methods that can be used to obtain utilities. These include visual analog scale, from using the standard gamble or time-tradeoff technique^3^ or from generic quality of life instruments such as the Health Utilities Index (HUI) Mark II/III^4^ or the EuroQol-5 Dimensions (EQ-5D).^5^ After obtaining utilities from any of the methods listed above, QALYs are calculated by multiplying the life years gained from an intervention by the utility weight.^3^

Many cost-effectiveness analyses are carried out alongside randomized controlled trials, where efficacy and cost data are collected prospectively.^6^ Trial-based cost-effectiveness analyses have appeal because of their high internal validity,^6^ however, such studies are expensive and time consuming and therefore are often not feasible to conduct. An alternative approach is to obtain efficacy and cost data from the secondary sources (i.e. published literature) and then input this data into a decision analytic model. A decision analytic model can be defined as a systematic approach to evaluate the impact of medical or surgical interventions on costs and other outcomes under conditions of uncertainty.^2^

Decision analytic models typically combine data from several sources such as randomized controlled trials, observational studies and expert opinion to produce detailed estimates of the clinical and economic consequences of different therapeutic alternatives.^2^ This permits the representation of the complexity of the real world in a more simple and comprehensive form and simplifying and evaluating complex clinical problems as an aid in the decision making process.^2^ The modeling approaches of decision analysis allow investigators to deal with other problems such as inadequate length of follow-up by using available data to estimate what will happen over the long term.^7^ Decision analysts can also examine a variety of cost assumptions and ways of organizing care and can calculate the sensitivity of their results to these alternate assumptions.^7^ The primary limitation of the decision analytic approach is that if its assumptions are flawed, it will not provide accurate results.^7^ At present time, there are a lot of controversies about decision analytic modeling, as many individuals believe that modeling is the perfect way to manipulate the results and therefore its credibility is limited.^2^ Decision analytic modeling is a potentially invaluable tool to assist the healthcare decision making process, although it is not a substitute for obtaining reliable and prospective evidence but rather a complement for real-time evaluation.^2^

When a cost-effectiveness analysis published in the orthopaedic literature shows that a new surgical technique is more cost-effective than the current surgical technique or standard of care orthopaedic surgeons need to consider the validity of the evidence before implementing the new treatment strategy. To make informed decisions orthopaedic surgeons can use a cost-effectiveness analysis to help them decide whether the new technique should be implemented, based on both efficacy and cost to the payer. The purpose of this article is to illustrate how to critically appraise a cost-effectiveness analysis using the principles of evidence-based medicine and the questions outlined in the Journal of the American Medical Association's Users' Guide to the Medical Literature.^7^

## CLINICAL SCENARIO

You are an orthopaedic surgeon who has a 56-year old male patient present with osteoarthritis of the hip. After carefully reviewing the patients x-rays and taking a detailed patient history which includes the failure of conservative treatment for hip osteoarthritis, it is evident that the patient has severe osteoarthritis of the hip and you recommend the patient to undergo total hip arthroplasty. At your hospital, the standard of care currently is metal-on-conventional ultra-high molecular weight polyethylene total hip implants. You are aware of the excellent long-term clinical and radiographic outcomes that have been reported using these bearing couples in total hip arthroplasty. However, there are always concerns regarding implant longevity and wear-induced osteolysis with conventional ultra-high molecular weight polyethylene as a bearing surface, particularly in younger, more active patients. A number of alternative bearings such as highly cross-linked polyethylene, second-generation ceramic-on-ceramic and metal-on-metal bearings are available for use. Although these newer bearings offer the potential to reduce implant wear, you believe that they may be associated with higher implant costs and the possibility for unintended consequences, including instability, impingement, ceramic fracture, material failure of cross-linked polyethylene and biological responses to metal ions, all of which could increase revision rates. As with all new health-care technologies, any potential benefits that could be derived from the use of these implants need to be considered in light of any additional clinical risks and economic costs that are associated with their use.

Your patient has previously expressed his desire to return to his previous level of activity (he was an avid runner). Prior to the patient's next appointment, when you plan to schedule his surgery, you decide to do a Pubmed search to determine if there are any recently published cost-effectiveness analyses comparing different bearing surfaces in total hip arthroplasty. If high quality evidence exists that demonstrates that an alternative bearing is a cost-effective procedure, you will discuss the results with your colleagues, operating room managers and hospital administrators.

## LITERATURE SEARCH

**Figure 1:**
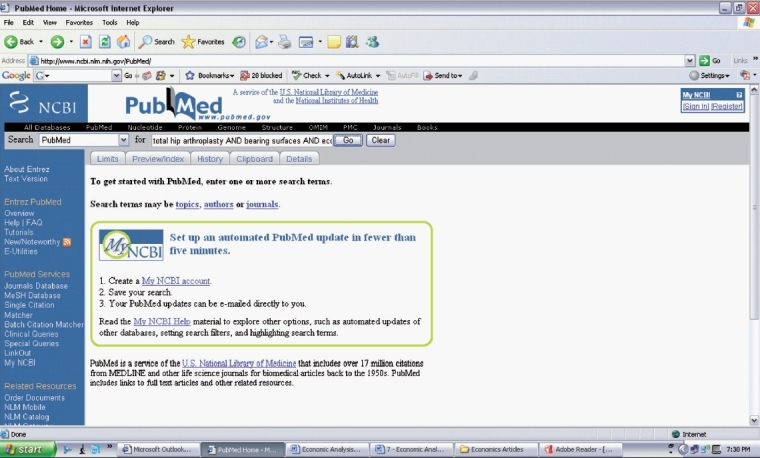
Search strategy

**Figure 2:**
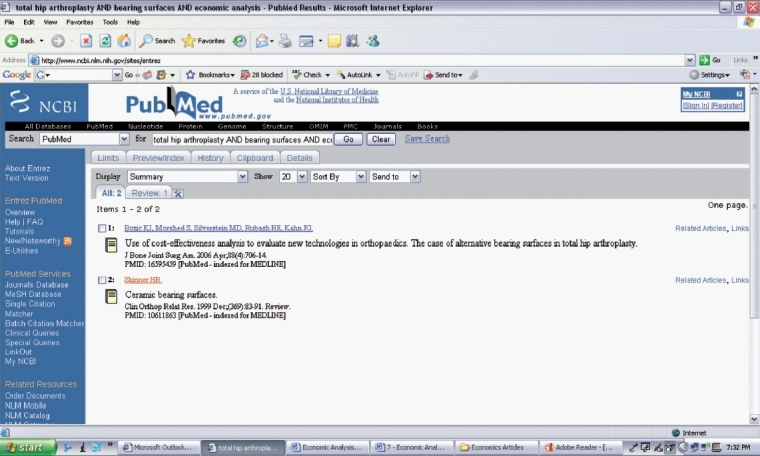
Search Results

**Figure 3:**
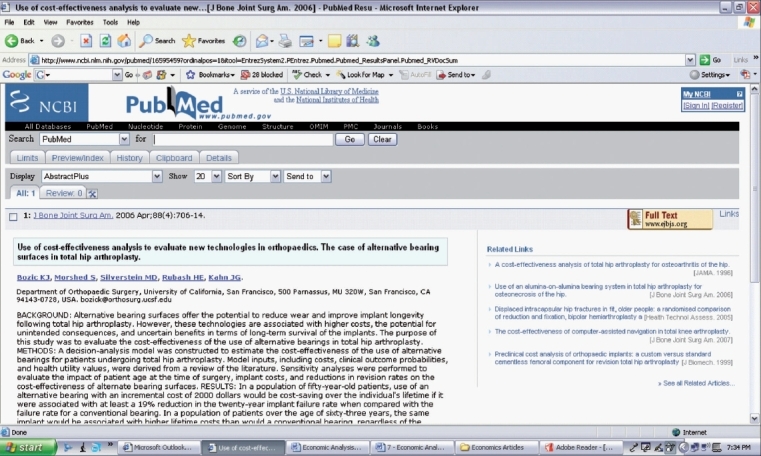
Relevant article

Using the *PICO* format, you develop your question for your literature search [Table 1]. You select the following key words from your research question (total hip arthroplasty AND bearing surface AND economic analysis) and enter them into the Pubmed search engine [Figure 1]. Your literature search yields two articles [Figure 2], one which addresses your research question [Figure 3].^8^ You decide to review and critically appraise the full article before deciding on which bearing surface to use for your total hip arthroplasty patient.

**Table 1:** PICO question for the literature search

P	Population	Male patients over the age of 50 with severe osteoarthritis requiring total hip arthroplasty
I	Intervention	Alternative bearing surfaces
C	Comparison	Traditional bearing surfaces
O	Outcome	Cost-effectiveness
Question		In male patients over the age of 50 with severe osteoarthritis requiring total hip arthroplasty, are alternative bearing surfaces more cost-effective than traditional bearing surfaces?

## CRITICAL APPRAISAL OF THE ECONOMIC ANALYSIS

This section will critically appraise the cost-effectiveness analysis based upon the questions asked in the Users' Guide to the Medical Literature [Table 2].^7^

**Table 2:** Critical appraisal questions to assessing the validity of a cost-effectiveness analysis

**Are the results valid?** Did the analysis provide a full economic comparison of health care strategies? Did the cost-effectiveness analysis consider all relevant patient groups, management options and possible outcomes? Does the cost-effectiveness analysis report results separately for patients who have different baseline risks? Did the cost-effectiveness analysis have a sufficiently wide viewpoint? Was clinical effectiveness established? Were costs measured accrately? Were data on costs and outcomes appropriately integrated? Were appropriate allowances for uncertainties made? Was the timing of costs and consequences considered?
**What are the results?** What is are the incremental costs and effect of each strategy? Do incremental costs and effects differ between subgroups? How much does allowance for uncertainty change the results?
**How can the results be applied to patient care?** Are the treatment benefits worth the risks and costs? In which settings could similar outcomes be expected? In which settings could similar costs be expected?

## ARE THE RESULTS VALID?

Examining the methods used is one of the most important steps in critically examining any scientific literature. The reviewer must understand how the results were obtained to determine the validity of the results. In economic analyses, the methods of the economic analysis must be critically examined along with the methods used to collect both the clinical and cost data. The following questions can guide a reviewer in determining if the results are valid.

### Did the analysis provide a full economic comparison of health care strategies?

A true economic analysis compares the clinical outcomes and the costs of at least two health care strategies such as two alterative treatment options, rehabilitation programs or two diagnostic tests. In other words, a full economic analysis must compare both clinical outcomes or efficacy as well as the costs associated with of each of the strategies being compared. If only the costs are compared, it is referred to as a cost-analysis. Cost-analyses do not take the patients clinical outcomes into perspective and are therefore not considered true economic analyses. There are several types of true economic analyses including cost-minimization analysis, cost-effectiveness analysis, cost-utility analysis and cost-benefit analysis. Cost-minimization analysis is a type of economic analysis that is used to compare cost differences among competing alternatives when these treatments are medically equivalent.^3^ A cost-minimization analysis should only be conducted in situations where the consequences of the alternative strategies are identical and therefore the only issue is their relative costs. Cost-utility analysis is a type of cost-effectiveness analysis. As described above, cost-effectiveness analysis measures effectiveness in terms of lives saved or limbs saved. Unfortunately these types of outcomes do not permit one to easily compare the benefits across different types of medical or surgical specialities. In a cost-utility analysis, the consequences or outcomes are expressed in terms of life-years adjusted by peoples' preferences; typically, one considers the incremental cost per incremental gain in quality adjusted life-years.^3^

A cost-benefit analysis is a form of economic analysis in which the costs and the consequences (including increases in the length and quality of life) are expressed in monetary terms.^3^ Cost-benefit analyses provide an estimate of the monetary resources consumed by each intervention under study compared to the value of resources the intervention might save.

Returning to our clinical scenario, Bozic's *et al.*, constructed a cost-effectiveness analysis using a decision analytic model (Markov model) to estimate the cost-effectiveness of the use of alternative bearings for patients undergoing total hip arthroplasty. A Markov model is used to examine scenarios that involve transitions between various states of health.^9^ A Markov model is a recursive model, allowing movement back and forth between points in the model. Advantages of a Markov model include the inclusion of the effect of time, thus identifying when events occur in the simulation, as well as providing a means by which to introduce more complex interactions between health states.^9^ In Bozic *et al.*'s Markov model, they inputted costs, clinical outcome probabilities and health utility values, which they derived from a review of the literature. Since both outcomes (QALYs) and costs are collected, Bozic *et al.*'s study is a cost-utility analysis, a subset of the cost-effectiveness analysis, which is considered a true economic evaluation.^8^

### Did the cost-effectiveness analysis consider all relevant patient groups, management options and possible outcomes?

In order for a cost-effectiveness analysis to be applicable to the clinical decision making process it must accurately consider all relevant patient groups, management options and outcomes. Patient characteristics such as age, gender, worker status and co-morbidities can often affect a patients clinical outcomes as well as the economic impact on the patient and society. In order to describe all therapeutic options available, information should be provided on the different study options as well as other relevant therapeutic options such as medications, surgical procedures or watchful waiting.^2^ Without considering all relevant management options, any decision plan will be incomplete. Additionally, if all possible outcomes are not considered, often the full clinical and economic impact may be missed.

Bozic *et al.*, based their analysis on a population of males who were 50 years of age with advanced osteoarthritis of the hip, who were candidates for total hip arthroplasty.^8^ This seems like a limited sample of the potentially relevant population (i.e. all patients requiring total hip arthroplasty) and consequently there may be limits to the generalizability of the results of the cost-effectiveness analysis. The authors did conduct sensitivity analysis varying ages over 50 years of age (see below for details on sensitivity analyses), which helps to strengthen the generalizibility of the results.^8^

The management options analyzed were hard-on-hard bearing (e.g, ceramic-on-ceramic or metal-on-metal) and a metal-on-conventional ultra-high molecular weight polyethylene bearing.^8^ The authors chose a metal-on-conventional ultra-high molecular weight polyethylene bearing as the comparator or referent group because it is the most widely used bearing surface as well as the bearing surface about which the largest amount of clinical and laboratory data have been published in the literature.^8^ A noted limitation of this study is the lack of high quality long-term data on current generations of alternative bearing implants.^8^ Laboratory data and early clinical data suggests the current generations of alternative bearings have lower wear rates, however long term data demonstrating any reduction in revision rates is lacking.^8^ Additionally, the performance of these alternative bearings (such as the biological responses to the alternative material wear particles, material failure or fracture rates and overall performance of implant) is relatively unknown.^8^ Alternately, there is a substantial amount of data on both short and long term outcomes of conventional ultra-high molecular weight polyethylene bearings.^8^ Another potential limitation is that all alternative bearings were grouped into one treatment strategy for both outcomes and costs.^8^

Bozic *et al.*, derived clinical outcome probabilities, including peri-operative mortality, death from other causes, complication rates and implant survival from the literature.^8^ Life expectancy and rates of mortality unrelated to the total hip arthroplasty were derived from age-specific actuarial life.^8^ The authors assumed no benefit of the alternative bearings in the first five years following primary surgery on the assumption that failures of any of the bearing surfaces could occur at any time.

Quality-adjusted life years were used as a measure of health utility and the authors derived utility values for each health state considered in the model from the literature.^8^ Obtaining utilities from the literature requires a number of assumptions to be made, which may limit the accuracy of the results.^8^

As described above, a number of assumptions are required in modeling, which potentially leads to uncertainty in the results. In addition, obtaining estimates of clinical outcomes and utilities from the literature as opposed to obtaining them by conducting a randomized controlled trial adds uncertainty to the analysis. The current method of dealing with these uncertainties and testing the accuracy of assumptions is through sensitivity analyses (described below).

### Does the cost-effectiveness analysis report results separately for patients who have different baseline risks?

The cost-effectiveness of any orthopaedic strategy will vary depending on the individual patients baseline risks. In other words, the costs and outcomes of a surgical intervention are related to the baseline risk of the condition under scrutiny. Patients who are at high risk will generally benefit more from a procedure than those at low risk.^10^ Examples of baseline risk include age, sex, stage of disease, co-morbidities, work status and activity level. Certain subgroups of patients may have a higher risk of a condition and are more likely to benefit from the new surgical intervention. Therefore, the ICERs may be dependent on the patient's ability to benefit from the surgical intervention. It is generally recommended that cost-effectiveness analyses report results separately for patients who have different baseline risks. For example, an economic analysis may present the ICER for males and then a separate ICER for females.

In the economic analysis conducted by Bozic *et al.*, the only different baseline risk that was analyzed was the patient's age.^8^ However, the age of patient may not be the most accurate measure of a patients baseline risks in patients undergoing total hip arthroplasty, as an individual's risk may be more related to their activity level.^8^ However, a patient's post primary total hip arthroplasty activity level is not only scientifically hard to quantify but also hard to clinically assume in a patient with debilitating osteoarthritis. Therefore, most investigators have used chronological age as a surrogate for physical activity level. Since this study was a decision analytic model, with the effectiveness data being abstracted from previously published literature, it may have been very challenging for Bozic *et al.* to appropriately adjust for all differences in baseline risk in their model.

### Did the cost-effectiveness analysis have a sufficiently wide viewpoint?

Cost-effectiveness analyses can be evaluated from several different viewpoints, most commonly the patient, hospital, third-party payer or society in general. In order for a cost-effectiveness analysis to effectively answer the questions raised, they must have a sufficiently wide viewpoint. It is generally recommended that the widest viewpoint feasible be used, such as the societal or the third party payer viewpoint.^10^

Bozic *et al.*, included all direct medical costs associated with the initial surgery and the treatment of any complications and follow-up care, including revision total hip arthroplasty.^8^ Since Bozic *et al.*, utilized a hospital's perspective; they did not consider the wider societal cost, all medical costs or the cost to the patient, which they mention as limitation of their analysis.^8^ They do justify their limited viewpoint by pointing out that it is likely that the other costs such as non-medical and indirect costs would likely parallel the results of this study as the probability of a revision would also increase the direct costs and rates of mortality while decreasing patient productivity.^8^ In addition, the results of the study may be different from the patient or societal perspective. In summary, an economic model should provide a societal perspective by taking into account all of the costs and consequences of the treatment including all healthcare costs, changes in productivity and impact on quality of life affecting all parties.^2^ Ideally, the approach should be transparently disintegrated into multiple viewpoints, including the patients' providers, healthcare system, major third party payers', hospitals and the primary decision maker to whom the study is primarily targeted.^2^

### Was clinical effectiveness established?

The preferred method of conducting a cost-effectiveness analysis comparing two orthopaedic procedures is one in which economic data are collected alongside a pragmatic randomized controlled trial. Pooling the results from many randomized controlled trials in meta-analyses help to increase generalizability because the pooled estimate of effectiveness is derived from a wider spectrum of patients than in one randomized controlled trial.^10^ While cost-effectiveness analyses that coincided with randomized controlled trials may provide a more accurate estimate of efficacy, the results may not be generalizable to the normal clinical population especially if there are strict inclusion and exclusion criteria as well as prescribed outcomes and follow-up. This can lead to higher compliance than the general population. Due to problems with feasibility of randomized controlled trials, many cost-effectiveness analyses establish the efficacy of the treatment strategies from previously published outcomes through decision analyses. Additionally, often long term clinical outcomes are not known, therefore a modeling study must be conducted. Modeling studies that can make projections of long term outcomes from short-term trial data relating to intermediate end points may be used to offset the problem of inadequate follow-up. A limitation to decision analytic models is that they must rely on the quality of the previous studies as well as the sometimes limited quality of the reporting of the results.

Bozic *et al.*, conducted a decision analytic model using previously published literature.^8^ In Bozic's study, the long-term clinical outcomes are only available with the metal-on- conventional ultra-high molecular weight polyethylene.^8^ Long-term clinical outcomes are relatively unknown for the alternative bearings; therefore a modeling study was undertaken.^8^ It is evident that a number of assumptions were made in determining clinical effectiveness due to the limited quantity and quality of the published literature.

### Were costs measured accurately?

The costs included in the cost-effectiveness analysis will be driven by the perspective selected by the investigators and the pathology under appraisal.^2^ When reporting costs, it is helpful to report the physical quantities of resources used by the competing strategies separately from their prices, as the price per quantity of an intervention varies among different locations, including provinces, states and countries.^10^ This will enable individuals in another jurisdiction to calculate the cost for their area of practice and reach a separate conclusion regarding cost-effectiveness of the new orthopaedic procedure. The methodology used to determine the units of healthcare utilization such as direct costs and working days lost should be described in detail.^2^ The decision about the inclusion of indirect costs in a model will rely on the condition under investigation, the perspective of the model and the intended audience of the publication.^2^ Another difficulty with valuing costs is that published charges of a particular surgical intervention may differ from the actual costs, depending on the bargaining power of health care institutions, third-party payers and the profit margin in a for-profit health care system.^3^

In Bozic *et al.*'s study, the costs of the primary and revision surgeries were based on actual hospital costs for the procedures. These data were reported in a previous published article by Bozic *et al.*^11^ The previous article was reviewed to determine how the costs were measured. Costs were determined based on the actual costs of the resource utilization at one hospital for 491 primary and revision total hip arthroplasties performed by two surgeons.^5^ The resource utilization was extracted from the hospital's administrative decision support database for inpatient hospitalizations.^5^ In critically, reviewing how the costs were measured, it is important to take into consideration that reporting the costs from one organization and only two surgeons adds cost biases. Each institution's costs will differ and reporting resource utilization such as operative time will vary based on individual surgeon's and hospital's practices therefore actual costs identified in this study may not be generalizable. Another limitation is that Bozic *et al.*'s study is that they only looked at hospital costs, many other direct medical costs were not considered, such as surgeon fees. The authors also state that they have included the costs for routine follow-up were determined from outpatient billing records.^8^ However, in reviewing the references in which these cost analyses were based, this was not clearly described.^11^

### Were data on costs and outcomes appropriately integrated?

On close scrutiny, some studies that purport to be cost-effectiveness analyses are not.^10^ A common error is to take the ratio of cost and effect of a surgical intervention and compare it to the ratio of the second intervention.^10^ In a cost-effectiveness analysis comparing two orthopaedic procedures, one is interested in determining the extra benefit that is gained from the extra cost. The appropriate method for integrating costs and outcomes is by calculating an incremental cost-effectiveness ratio (ICER). The numerator of the ICER is the marginal difference of the mean cost of each intervention and the denominator is the marginal mean difference of the effectiveness.^3^ The formula for calculating an ICER is = [cost_experimental_ - cost_traditional_]/[effect_experimental-_ - effect_traditional_].^3^

There are two scenarios when calculating an ICER is not necessary. The first scenario that does not necessitate the calculation of an ICER occurs when one surgical intervention is both less expensive and more effective. In this situation the procedure is dominant and this is referred to as a win-win scenario. Conversely, if a new procedure or technique is more expensive and less effective, this is referred to as a lose-lose situation and there is also no need to calculate an ICER.^10^

For the baseline analysis, Bozic *et al.*, present value for the lifetime incremental costs per QALY gained following total hip arthroplasty in a fifty-year-old patient treated with an alternative bearing couple with an incremental cost of $2,000 compared to the value for a patient of the same age treated with a metal-on-conventional ultra-high molecular weight polyethylene bearing couple.^8^

### Were appropriate allowances for uncertainties made?

There is almost always uncertainties in economic analyses, especially in modeling where a large number of assumptions are required. Uncertainties can result from the estimation of inputs or from methodological issues. Often data available to the investigators are secondary, not obtained from randomized controlled trials but from studies of lesser evidence value.^10^ In addition, these studies were not designed as economic evaluations and may not have recorded and presented all required data to conduct a cost-effectiveness analysis. In such a situation the uncertainty in the estimation of both costs and consequences can be problematic. To allow for uncertainties in economic analysis, investigators typically conduct sensitivity analyses. In sensitivity analysis, the uncertain variables are examined across a range of values to asses their impact on the study results.

In the decision model conducted by Bozic *et al.*, multiple sensitivity analyses were conducted assessing variations in age, incremental implant costs and variable reductions in the probability of implant failure at 20-years.^8^ Age was varied from 50 to 75 years of age in 5 year increments, incremental implants costs varied from $500 (assuming lower cost bearing surfaces such as highly crosslinked ultra high molecular weight polyethylene) to $4,000 (for more costly bearing alternatives such as diamond-on-diamond bearings that are being explored).^8^ With the great uncertainty of the failure rate of alternative bearing surfaces, the reduction in the probability of implant failure at 20 years was varied from 0 to 70 percent.^8^

### Was the timing of costs and consequences considered?

The analytic time horizon should extend far enough into the future to capture the major clinical and economic outcomes of the alternatives under assessment.^2^ The selection of the time period will depend on the nature of the clinical question, the period of time required to achieve therapeutic effectiveness and the time required to detect adverse events and complications, including long term complications.^2^ Typically, decision analytic models cover the period from the initial treatment until recovery or death, while in a Markov model, ideally a lifetime follow-up is recommended.^2^ Bozic *et al.*, clearly specify their time horizon as the remaining life expectancy of the patient.^8^ This is the appropriate time horizon to use given the clinical question and the study design.

It is generally accepted that people prefer to obtain the benefits of an intervention sooner and postpone the costs for the future. Discounting is the valuation of costs and consequences over time.^3^ In cost-effectiveness analyses, it is normally acceptable to discount costs and benefits occurring in the future to present values.^3^ The general agreement on the discount rate varies between three percent and five percent.^12^

Based on the recommendations of the Panel on Cost-Effectiveness Analysis in Health and Medicine,^13^ Bozic *et al.*, discounted all costs and health benefits at a constant rate of 3 percent per annum in order to determine the net present value of costs and quality-adjusted life years gained with each treatment strategy.^8^

## WHAT ARE THE RESULTS?

Once the methodology for obtaining the results has been reviewed, the results themselves must be critically examined. This section provides questions to ask when determining what are the results of the economic analysis.

### What are the incremental costs and effect of each strategy?

In reviewing the results of a cost-effectiveness analysis, one should first look for the ICER. Incremental cost effectiveness ratios report the additional cost per additional unit of effectiveness measured as quality adjusted life years and they are generally expected in a cost-effectiveness analysis. Incremental cost effectiveness ratios compare the incremental cost of the best known alternative to the new alternative.

Bozic *et al.* should have presented an ICER demonstrating the cost per QALY gained when using alternative bearings in total hip arthroplasty.^8^ This figure was not clearly presented in their article. Instead, Bozic *et al.*, present the following results “in a population of fifty-year-old patients, an alternative bearing with an incremental cost of $2,000 (the average incremental cost associated with a hard-on-hard bearing couple) would need to be associated with at least an 18.7 percent reduction in the probability of implant failure at twenty years, when compared with the probability of failure of a conventional metal-on-ultra-high molecular weight polyethylene bearing, in order to be cost-saving over the lifetime of this group of patients”.^8^

### Do incremental costs and effects differ between subgroups?

Patients with different baseline risks will most often be affected differently by the incremental costs and effects. If there is any existing evidence that the results of the study may be different in a subgroup of patients, it is recommended that different analyses be performed with each subgroup to detect any economically significant differences.^2^ Examining different subgroups may have major implications on the cost-effectiveness. For example, different subgroups of patients (gender, age, comorbidities) may have different rates of treatment failures and mortality.

In this study, the incremental costs and effects were compared across multiple ages.^8^ As life expectancy and rates of mortality change with patient age, this in turn affects the cost-effectiveness of an alternative bearing surface at differing ages. For example, an alternative bearing with an incremental cost of $2,000 would be cost-effective based on a willingness-to-pay of $50,000/QALY with just a 3.8 percent reduction in the twenty-year probability of implant failure in a 50 year old patient.^8^ However, the same implant in a 70 year old patient would require a 45.5 percent reduction in the twenty-year probability of implant failure to be considered cost-effective.^8^

### How much does allowance for uncertainty change the results?

Decision analytic models of cost-effectiveness analysis use multiple assumptions when the outcomes and the resource consumption associated with a treatment strategy are unknown. In each model there will be uncertainty about the correct value for multiple variables.^2^ It is important for these uncertainties to be addressed, especially how these uncertainties change the results.

One method to account for uncertainties is to perform a sensitivity analysis. A sensitivity analysis can help determine the influence of different factors on the results of the investigation. There are several different types of sensitivity analyses including a simple univariate analysis (one-way analysis), simple multivariate analysis, threshold analysis, analysis of extremes and probabilistic analysis (Monte Carlo simulation).^2^ In a one-way sensitivity analysis each factor is examined separately to determine how varying the factor affects the results. However, often there are multiple uncertainties. With multiple uncertainties, one-way analyses often underestimate the effects on the results. In multiple-way sensitivities, two or more factors can be examined simultaneously. The choice of variables on which a sensitivity analysis is performed should be justified and the rationale for the interpretation of the results of such an analysis should be clearly defined.^2^ It is also advisable to include the best and worst case scenarios or values of the variables and the values of the confidence intervals if available.^2^

In this article, multiple sensitivity analyses were conducted using the Markov model to assess the impact of variation of age at the time of primary total hip arthroplasty, incremental implant costs and reduction in the probability of implant failure at twenty years on the relative cost-effectiveness and the lifetime cost-savings associated with each treatment.^8^ Bozic *et al.*, varied the incremental implant costs of the alternatives bearings from $500 to $4000 in $500 increments.^8^ Additionally, they varied the reduction in the probability of implant failure at twenty years from 0 percent to 70 percent.^8^ They based their thresholds on willingness-to-pay $50,000 per QALY gained.^8^ Their analysis showed that in a 50-year old male, an alternative bearing couple with an incremental cost of $500 would be cost-effective on the basis of willingness-to-pay of $50,000 if the incremental reduction in the probability of revision at twenty years was ≥1.1 percent.^8^ On the other hand, an alternative bearing implant with the incremental costs of $4,000 would require a reduction in the probability of revision at twenty years of ≥7.5 percent.^8^ Overall, sensitivity analysis determined that as the incremental implant costs increased, the age threshold of cost-savings decreases and a greater reduction in the probability of implant failure is required to justify the additional costs of the implant.

## HOW CAN THE RESULTS BE APPLIED TO PATIENT CARE?

The results of a good economic analysis should be able to help determine the optimal treatment strategies. After carefully reviewing methodology and results to ensure that the results can be generalized to a particular jurisdiction, the results can then be applied to patient care. This section provides several questions to ask when determining if the results of an economic analysis will be applicable to a different jurisdiction.

### Are the treatment benefits worth the risks and costs?

“Do the benefits outweigh the risks?” is one of the most important questions to ask before implementing any new treatment strategy into practice. Both effectiveness and costs need to be included in these risks. When a new treatment has been shown to be both less costly and more effective, the decision is easy and is said to be strongly dominant. Conversely, the decision to reject a new treatment that is both less effective and more costly is also an easy one. When one of either the effectiveness or costs of the treatments are equal it is called weak dominance. This can be weak dominance to accept the treatment with either equivalent costs but better effectiveness or lesser cost with equal effectiveness. Weak dominance to reject occurs with either greater cost with equivalent effectiveness or equal costs with less effectiveness. However, circumstances of non-dominance, such as no difference in effect or cost or additional effectiveness but also additional costs, require incremental costs calculations.

Many new treatments are both more effective and more costly, which presents a challenge to decision makers in deciding whether to adopt the new technology. In a cost-utility analysis it is generally accepted that if an intervention has an ICER below the threshold of $20,000 per QALY gained, there is a strong indication for its acceptance. Alternatively, if the ICER is above the threshold of $100,000 per QALY gained, there is an indication for its rejection.^14^ There is much discussion in the literature regarding the interpretation and application of the ICER in cost-utility analyses.^15^ The quantitative thresholds set by Laupacis *et al.*, in 1992 are criticized for being arbitrary and outdated, although they remain in frequent use.^16^–^18^ The National Institute of Health and Clinical Excellence of the British National Health Services uses ?20,000 per QALY gained as their ICER threshold for the acceptance of new technologies.^19^,^20^

Unfortunately Bozic *et al.*, do not present an ICER showing the cost per QALYs gained in their publication. Instead, they present cost-saving data, which is also relatively simple to interpret. Assuming that the alternative bearings do show a reduction in implant failure at 20 years of at least 18.7%, according to this study an alternative bearings with an incremental cost of $2,000 would be cost-saving in 50 year old, male patient. However, the same implant would not be cost-saving for the patient over the age of 63 years regardless of the reduction in implant failure. The same alternative bearing implant with an incremental cost of $2000 would be cost-effective in a 50 year old, male patient based on willingness-to-pay $50,000 per QALY with only a 3.8% reduction in implant failure at 20 years. While to be cost effective in a patient over 75 years, the reduction in implant failure at 20 years would have to be at least 45.5%.

### In which settings could similar outcomes be expected?

If in critically evaluating an economic analysis that shows a new treatment is cost-effective, one needs to determine if those results would apply to the patients in their practice or hospital. The clinical evidence on which the cost-effectiveness is based is often from clinical trial data. In clinical trials, a structured treatment protocol is usually followed, often with strict inclusion and exclusion criteria that can generally lead to a highly specific and compliant patient population. Therefore, the efficacy of a treatment in a clinical trial may not exhibit the same effectiveness in the general orthopaedic population. As mentioned above, the data included in decision analytic models come from many different sources, which may or may not be similar to the patients in your jurisdiction.

To determine whether patients in your jurisdiction can expect similar outcomes, the patient population that the clinical outcomes were based needs to be compared to the patients in your practice or hospital. Factors such as gender, age, comorbidities, economic and social status and cultural differences can lead to variations in patient outcomes. Additionally, to expect similar outcomes, one must have similar treatment methods to those analyzed in the economic analysis. If the economic analysis is calculated from the literature or historical controls, the referenced literature must be examined in order to determine if both the patient population and the treatment methods are comparable to your practice.

In their study, Bozic uses previously published clinical and health related quality of life utilities to create their model, using values obtained from conventional bearings. It is important to review the referenced studies and assess whether the patients included in these studies are similar to your patients. In addition, one needs to assess whether the implants used in these studies similar to the ones available in their operating suite. In addition, with 20 year revision rates based on published literature, the implants used in those studies may not be comparable to the implants used today.

### In which settings could similar costs be expected?

Just like patient outcomes vary between different populations, costs also can very between populations, treatment providers and geographical locations. Again, treatment practices may be different requiring different resources. Additionally, the costs of resources may be different from those in the study. In Bozic *et al.*' study, the costs of resources were from a single institution and from only two surgeons. This study focused on the inpatient hospital costs and did not include all direct medical costs. To determine if costs provided in the study are similar to the costs in your jurisdiction, there are a number of questions to ask. Could the inpatient costs and resources vary in your practice? There are many variables in resources in your practice that may be different from those in San Francisco, California, USA. Are post-operative medication regimens similar? Are rehabilitation time and resources similar? Are there differences in operating room times and staff? All of these can greatly affect the costs between different institutions and different jurisdiction.

#### Resolution of the clinical scenario

Although models are increasingly being used in cost-effectiveness analyses in surgery, there are several limitations and problems with their use, primarily due to the sources used and the assumptions included their structure, the correct interpretation of sensitivity analysis, the degree of transparency of the model and its proper validation in the usual healthcare practice.^2^ Decision analytic approaches, such as the one utilized by Bozic *et al.*, are prone to bias because they gather information for many different sources.^2^ Another common problem with models is their transparency, as potential for manipulation is high.^2^

Returning to our clinical scenario, your patient's age and activity level place him at a high risk of revision with a conventional bearing total hip arthroplasty. Using Bozic's cost-effectiveness analysis based upon a decision analytic model, an alternative bearing surface could be cost-effective in your patient. However, there is still little clinical evidence to show definitive twenty year revision rates or QALYs gained with alternative bearings. In addition, Bozic's study is a decision analytic model with multiple assumptions. At this time you do not believe that there is sufficient evidence to warrant a change in your surgical practice. You will however continue to look in the literature for additional research supporting Bozic *et al.* 's conclusions.
